# Perceptions and Portrayals of Skin Cancer among Cultural Subgroups

**DOI:** 10.1155/2014/325281

**Published:** 2014-01-28

**Authors:** Stephanie Kelly, Laura E. Miller, Ho-Young Ahn, J. Eric Haley

**Affiliations:** ^1^North Carolina A&T State University, USA; ^2^University of Tennessee, USA; ^3^Southern Connecticut State University, USA

## Abstract

Health communication scholars have a responsibility to be certain that both healthcare practitioners and government agencies accurately communicate health information to the public. In order to carry out this duty, health communication scholars must assess how messages are being received and if they are being received at all by the public. This paper details a two part study which assesses this phenomenon within the context of skin cancer. Study 1 utilized 29 in depth qualitative interviews to identify subcultures among college students whose communication puts them at risk for skin cancer by encouraging poor sun exposure behaviors. The results indicate that farmers, African Americans, and individuals who regularly participate in outdoor athletics are at risk groups. Study 2 reports a content analysis of the known population of skin cancer Public Service Announcements (PSAs) available via the internet in 2013. The aforementioned groups were not present in any of the PSAs. Detailed results and implications are discussed.

## 1. Introduction

Health communication scholars have a responsibility to be certain that both healthcare practitioners and government agencies communicate health information to the public accurately [1]. In order to carry out this duty, health communication scholars must assess how messages are being received and if they are being received at all by the public. This study explored the sun care behaviors of individuals as they are influenced by health education, family practices, and cultural beliefs. More specifically, the current two part study investigated whether the communication among the subcultures interviewed encourages the formation of high risk/marginalized groups in regard to sun exposure and sun care, and whether these high risk/marginalized groups were targeted in government sponsored healthcare promotion campaigns.

High risk/marginalized groups include demographics that “often have difficulty affording or gaining access to healthcare, and they are often targets of discrimination due to the prevalence of ageist, sexist, and racist beliefs in our culture” [2, pages 216-217]. Thus, these groups are historically composed of individuals in the lower socioeconomic class and come from homes with less means to seek out higher education or healthcare. Individuals who have a lower level of education are likely to be less educated about health risks and are less likely to seek out health information [3]. As a result, marginalized groups are composed of the individuals who are least likely to seek out health information and least likely to have the means to regularly visit a healthcare provider. Because high risk groups are those least informed about preventative healthcare, they are groups that necessitate outreach through Public Service Announcements (PSAs).

PSAs are a form of government funded social marketing campaigns [4]. The primary purpose of PSAs is to provide information to or influence the behavior of specific targeted audiences [5]. Research has indicated that the primary focus of an advertisement, such as a PSA, the element that truly captures a consumer's attention and the element from which a consumer determines whether the message is relevant to them personally, is the pictorial element [6, 7]. Therefore, the picture featured in a skin cancer PSA is most likely to indicate to the consumer whether the PSA is relevant to them. More specifically, a person is more likely to pay attention to an advertisement in which they see someone that reminds them of themselves.

The overarching purpose of the present study is twofold. The first purpose is to identify groups among whose cultural communication encourages risky behaviors regarding sun exposure. Assuming such groups exist, the second purpose of the study is to assess the presence of the identified groups in current PSAs.

### 1.1. Skin Cancer

The second leading cause of death in the United States as of 2010 is cancer, accounting for 567,628 yearly mortalities [8]. Skin cancer is the most common type of cancer [9–12]. The most lethal form of skin cancer, metastatic melanoma, typically leads to death [9, 13], with 8,461 cases of melanoma related deaths in the United States in 2010 [9]. Less aggressive forms of skin cancer which typically manifest as thin, stage one lesions are usually treatable. Avoiding ultra-violate radiation (UVR) is the most effective way to prevent skin cancer [9, 10].

Particularly for women, exposure to ultra-violet radiation up to age 20 is linked more closely to developing melanoma than exposure after age 30 [14]. Previous research has indicated that farmers [11, 15–17], African Americans [18], and athletes who participate in outdoor sporting events [19, 20] are at risk groups who are less aware of the salience of preventative skin care. The overall survival rate of melanoma among African Americans is only 77% compared to the 91% survival rate for Caucasians [21]. Additionally concerning for African Americans is that skin cancer is more aggressive when it manifests in many African Americans than when it manifests in Caucasians [22]. Moreover, Asians and African Americans are more likely than Caucasians to present with an advanced case of melanoma at the time of diagnosis [21].

No one is immune to skin cancer [21]. Despite the fact that the negative effects of excessive sun exposure are prevalent in the media, tanning is fashionable within American culture, particularly among young women [23]. As a result, many individuals who do not recognize the salience of preventative skin care behaviors are putting themselves into high risk groups for developing skin cancer because they do not recognize the need to change their current skin care habits [24].

### 1.2. Information Seeking

If individuals do not realize that they are at risk for developing skin cancer, they are unlikely to seek out information on preventative behaviors or actively process information presented to them on these behaviors. The phenomenon of information seeking in the face of cancer is a topic that has intrigued health communication scholars for years [25]. Although it is common for patients to want to know about their disease, the amount of information that individuals want to know varies greatly [26]. When faced with severe illness, some people actively seek out information to decrease their uncertainty because their current level of uncertainty causes distress [27]. However, when information seeking uncovers contradictory information or leaves the seeker with a sense of hopelessness, then the act of seeking out additional information becomes counterproductive as it has led to a higher degree of uncertainty [28]. In this case, avoiding information can act as a buffer from reality [29]. Therefore, this act of avoiding information can be used as a method of *information management* in that the avoidance maintains a person's mental health [30].

Given the numerous preventative measures that can be taken to avoid cancer [31], it is more ideal for an individual to seek out information on preventing the development of skin cancer rather than waiting until a time in which it has become necessary to seek out information on treating or living with it. Although no one is immune to skin cancer [32], most people choose to ignore information about cancer until it becomes relevant to their daily lives [25]. According to Case et al. [29], salience, the act of cancer becoming relevant to a person's needs and concerns, is a central motivation behind information seeking regarding cancer. Study 1 seeks to identify the perceived salience of skin cancer among subcultures.

## 2. Study 1

Undergraduate college students are often in the unique position of making their own health decisions for the first time; thus, these individuals are forced to evaluate their prior beliefs and behaviors regarding healthcare in order to form their own beliefs and make independent decisions. Communication and culture reflect and mold one another [33]. Therefore, college students, who are likely to be evaluating (or to have recently evaluated) their healthcare norms derived from their upbringing, thereby making cultural communication regarding health beliefs salient, were chosen as the sample for this study. To explore this phenomenon, the following research questions were posed:

RQ1: Are there groups of college students whose communication within self-identified subcultures indicate that they are at risk regarding sun exposure and sun care?

### 2.1. Method

#### 2.1.1. Participants

Twenty-nine undergraduate students from a moderate sized southeastern university participated in this study (14 males and 15 females). Participants were between 18 and 25 years of age. These participants were recruited in exchange for five points of extra credit in an introductory public relations course that was not taught by the researchers.

#### 2.1.2. Procedures

The interviews were semistructured. An interview guide was utilized. Sample items included “Tell me about your skin care bahviours,” and “Do you think about the sun's influence?” Participants were encouraged to lead the interviews, narrating the rationale for their behaviors in the fashion that made most sense to them. Redundancy occurred at 20 interviews; however, interviewers collected 9 additional participants to verify that redundancy had indeed been reached. All interviews were performed by the same two researchers for consistency.

#### 2.1.3. Data Analysis

When interviews were complete, the interviewing researchers transcribed the interviews. A thematic analysis was performed on the transcripts. Interviews were searched for indications of ethnicity, self-identified cultures (i.e., families, organizations, teams, occupations, etc.), activities, and sun care. Unfortunately, not all participants self-identified their ethnicity. As such, it was not always accounted for. The themes that arose involved race, occupation, and participants' levels of physical activity. Individuals in subcultures that revolved around physical activity were categorized as an outdoor athlete, indoor athlete, or outdoorsman/outdoorswoman. However, some participants self-identified themselves with more than one of these groups. This was resolved by categorizing participants according to the activity that they related to most highly. For instance, if a participant commented that sometimes they fish, but elaborated on playing baseball, then even though fishing falls under the outdoorsman/outdoorswoman category, the participant self-identified more thoroughly with outdoor athletics, meaning that their identity is more heavily influenced by outdoor athletics.

### 2.2. Results

The findings indicated that there were three subcultures among our participants whose group communication encouraged poor sun exposure behaviors in regard to wearing sun block. Among this group were farmers, African Americans, and outdoor athletes. The following paragraphs represent the themes as well as direct quotes from the interviews to support their existence. To reiterate, since the interviews were semistructured and the interviewees were encouraged to guide the interviews, the quotes supporting each theme were not always responses to a direct question. Interviews began by prompting participants to talk about themselves, particularly about hobbies and favorite activities. When participants brought up a reference to tanning or being outside, the interviewer prompted the participant by asking them to elaborate on that topic. The following quotes are the result of such prompts.

#### 2.2.1. Farmers

Not all of the individuals who fall into the “farmers” category actually self-proclaimed themselves to be a farmer. Yet, each individual at least made a special point to mention how much they valued spending time on the family farm as a child. Some participants even used this time spent on the family farm as justification for poor sun exposure behaviors now.

F1: * I think my grandma, she has a farm out back she has a garden and doesn't even go to the store to get her vegetables she tries to tell me… I've never burned, I stay in the sun all the time. None of my family has. My grandmother is 80 and her skin looks great… I don't even use sun block.*
F2: * I like outdoors: hunting, grew up on a family farm, so I like being back there. I try to be somewhat concerned with it because my dad has some skin cancer spots, so I try to… I don't know… I don't wear sunscreen but I should. I guess I'm not as concerned as I should be.*
F3: * Not really because I've worked on a farm my whole life… my family has a farm. So, I'm pretty much going to have cancer so I'm not really worried about it, so I mean… Occasionally, but it's only when I know I'm going to be out there for long periods of time without my shirt on, but if I'm just going to wear cut off sleeves and a t-shirt then no way because my skin is too… used to the sun already. I just take one time to get burnt and I'm good for the rest of the summer. Like, arm-wise because my chest and back are pretty fair still so yeah, I would wear sunscreen on that.*

As such, 100 percent of the participants who mentioned farming in any way self-proclaimed that they do not regularly use sun block. This rationale was based on a lifetime of observing family members who did not wear sun block as well as a fatalistic mentality that given the amount of sun exposure they had experienced up to this point in their life, changing their behaviors at the present time seemed unlikely to reduce their likelihood of developing cancer.

#### 2.2.2. African Americans

Another subculture that expressed poor sun care behaviors were the African American participants. Only three individuals identified themselves as African Americans among the participants. However, when asked about their skin care habits, their responses were uniform.

AA1: * No, I don't. Not at all because I don't even like to go outside that much.*
AA2: * I guess as an African American, I don't even use sun block.*
AA3: * Not really… I just try to stay indoors when it's really hot and try to go outside in the evening once it's cooled off.*

It is particularly concerning that one of the participants explicitly stated that he did not use sun block because he is an African American. Even the participant who tried to avoid being outdoors in the summer to avoid tanning did not report using any form of preventative skin care when outside.

#### 2.2.3. Outdoor Athletics

Outdoor athletes were classified as any person who plays team or recreational sports outdoors including soccer, baseball, softball, football, or running track. This did not include leisure sports such as hiking or fishing. Although each athlete recognized the need to wear sunscreen, it was not a habit he or she proclaimed to practice.

OA1: * Swimmer: Well, I have terrible habits. When it comes to honesty, I spend every day when I work in the summer… So I'm on or around the water with direct exposure to the sun five months out of the year and never use sun screen.*
OA2: * Softball player: I do, when I'm playing, put sunscreen on my face and not wear makeup to clog up my pores, but if I'm laying out I usually just wear like SPF 4 and don't lay out for more than an hour and a half.*
OA3: * Soccer player: I've gone to the tanning bed for 3 years and I usually only go for a couple weeks a year. But I play soccer and so I am in sun a lot with that and my face always gets burnt and I know that I need to put sun screen on but I don't really think about it and I don't want it to sweat in my eyes. I know it's bad for me but I never put it on. And in the summer I'm in the sun because I lay out and stuff.*
OA4: * Baseball player: I don't see a problem with going to get sun if you do it properly. You know if you use your suntan lotion and stuff?… Yeah, sometimes, but I'll be the first to tell you I'm not the best at that, I could do a better job… I do that quite a bit and I could do a better job with my skin and uh I seldom, seldom use sunscreen.*

At first, these themes could be construed to imply that people who play team sports in general practice poor sun exposure behaviors. However, this notion was contradicted by the presence of indoor athletes and outdoorsmen/outdoorswomen.

#### 2.2.4. Indoor Athletics

Indoor athletes were categorized as anyone who claimed to enjoy playing sports indoors, particularly basketball players and indoor track runners. These individuals self-reported that they wore sun block on a regular basis.

IA1: * Basketball player: As far as being active, I try to be active with sports, basketball. I try to maintain a healthy lifestyle—work out every day, or try to… Yes, because I don't want to get sun burnt being out in the sun too long playing sports. You don't want to get sun burnt and be like, peeling and stuff. That's not a good feeling in the shower.*
IA2: * Indoor runner: I like to run. So, I mean… Sometimes I do sit ups and stuff like that, but I don't do major workouts, but I like to run a lot. Yeah, on the track… The beach? I always put on sunscreen. You know, that's… I have to do that, because it protects your skin and everything. So I've always done that since I was a child, just put on extra sunscreen.*

To further support the notion that not all athletes have poor tanning behaviors, a third group of athletes emerged from the interviews.

#### 2.2.5. Outdoorsmen/Outdoorswomen

Outdoorsmen/Outdoorswomen included anyone who rather than identifying a sport qualified that they enjoyed nature through hiking, backpacking, rock climbing, or fishing. This group reported being very aware of the sun, with some participants even volunteering their armor of protective clothing.

OD1: * I go backpacking and hiking. I used to go rock climbing, but I don't do that anymore. Canoeing, I've been backpacking in Mexico, canoeing in Canada… When you're backpacking a mile above sea level the air is thinner and you burn more easily so we're always wearing a hat. I even have one of those goofy hats that hangs down and keeps your neck from burning and we usually use sunscreen too. Going to the lake too I definitely always use sunscreen. I got burnt really bad a couple of times when I was younger so I'm definitely a lot more careful just because of the pain and discomfort. Getting burnt over your whole body is terrible!*
OD2: * I like to fish, real big fisherman and angling… Yeah, I hunt. Well, some duck hunting, yeah… Yeah, I always put on sunscreen.*
OD3: * I like to be outside. I like to go outside. All that… Um… Well, earlier this summer when I was at the beach I went to the beach like every day and laid out but I always used sunscreen. And then now, I try to go to the pool sometimes—like twice a week maybe, but I still use sunscreen though.*

The presence of outdoorsmen/outdoorswomen's and indoor athletes' health conscious sun exposure behaviors implies that athletes who play team sports outdoors are the only emergent group of athletes who practice poor sun exposure behaviors.

### 2.3. Study 1 Discussion

The findings of this study imply that undergraduate farmers, African Americans, and outdoor athletes exist within subcultures whose communication encourages poor sun exposure behaviors. This places these students into high risk groups. These findings were supported in that African Americans are known to be a marginalized group, who are reported to be low information seekers regarding health information [2]. In further support of these findings, Robinson et al. [11] identified soccer participants and farmers as at risk groups of adults regarding skin cancer. Other research has also given attention to sun care within the outdoor athletics culture, but this research focused more on the exposure of parents and coaches than the athletes themselves [34]. Including parents and coaches in such studies of adolescent sports is important because they make up part of the culture, but there has been insufficient investigation of college athletes and sun care behaviors.

Robinson et al. [11] also found that both farmers and soccer participants were more likely to participate in preventative sun exposure behaviors when they had been counseled in how to do so by their healthcare provider. It is the responsibility of health communication scholars to ensure that healthcare providers are sharing such information with their patients. It is also health communication scholars' responsibility to make preventative skin cancer behaviors known to these high risk/marginalized groups [1]. However, presenting information does not necessarily imply that the information will be seriously considered. It is important to make sun exposure prevention and skin care maintenance a salient topic to these groups of students.

## 3. Study 2

The results of Study 1 have indicated that African Americans, farmers, and outdoor athletes are groups of college students who exist in subcultures that are unaware of the salience of skin cancer and/or preventative care. PSAs are necessary to further drive home the importance of preventative skin care behaviors. The purpose of PSAs is to enlighten people through exposure to beneficial, unbiased information, often pertaining to health concerns, and direct them towards preventative health behaviors [35]. This begs to question whether these groups are being targeted in skin cancer PSAs. Study 2 seeks to assess the target audience of the PSAs by specifically answering the following research questions.

RQ2: Who is portrayed in current skin cancer-related PSAs?
RQ3: What activities are the individuals featured in current skin cancer-related PSAs engaged in?
RQ3a: If the individuals featured in the skin cancer-related PSAs were not engaged in an activity, are they wearing apparel that is indicative of a particular occupation or activity?
RQ4: Who is the target audience of current skin cancer-related PSAs?
RQ5: Where are the skin cancer-related PSAs being posted/shared?

### 3.1. Method

#### 3.1.1. Sample

Because the internet is the most frequently accessed resource for health information [36], the present study looked to the internet as a source of still image skin cancer PSAs. Because the rate of information upload on the internet is astronomical, for the sake of manageability, the present study limited itself to capturing PSAs available on the first day of each month for one year. PSAs were collected monthly from websites for The American Cancer Society, American Academy of Dermatology, The Melanoma Foundation, The Skin Cancer Foundation, Sun Smart, and The World Skin Cancer Foundation. Google and Google Images were also checked each month under the search terms “skin cancer PSA” and “skin cancer public service announcement.” The researchers believe this to be the full population of internet PSAs available during 2013 (*N* = 43).

#### 3.1.2. Coding

The PSAs were coded by two of the researchers for sex (male, female, both, and indeterminate); ethnicity (Caucasian, African American, Asian, multiple races, and other); presence of a beach (present, not present), occupational activity (present and not present), or athletic activity (present and not present); age (0–8, 9–16, 17–25, 26–33, 34–42, 43≤, or indeterminate); and target audience. To establish inner coder reliability, five of the PSAs were randomly selected and pilot coded by the researchers separately. The researchers reconvened and discussed their differences until they reached consensus. The data set was then independently coded by both coders. Inner coder reliability was calculated by Cohen's kappa. Reliability was deemed accepted if and only if *k* ≥ .9. Reliability was acceptable in all categories with overall *k* = .991.

### 3.2. Results

Research question 2 asked who was featured in current PSAs. Of the 39 PSAs coded, only 33 (85.6%) featured a pictorial element which included a person. Of these 33 PSAs, 20 featured only females, 4 featured only males, 2 featured both males and females, and 7 featured models of indeterminate sex. One hundred percent of the people portrayed in the PSAs were Caucasian. Age was the most diverse characteristic displayed within the sample; 6 of the people were no older than 8 years old, 8 were between 16 and 25 years old, 6 were between 34 and 42 years old, and 13 were of indeterminate age because their faces were not visible.

Research question three asked what activities individuals depicted in PSAs were engaged in or what activity-related apparel they were wearing. None of the individuals depicted were working or wearing apparel indicative of an occupation nor were they engaged in athletics or wearing athletic apparel. However, 10 of the people in the PSAs were shown at a beach.

Next, research question four addressed the target audience. The purpose of this question was to identify if the tanner was being addressed in the PSA or someone connected to the tanner. Among all sampled PSAs, 34 targeted the tanner and 5 targeted someone connected to the tanner, specifically the parents of the tanner.

Finally, RQ5 asked where the PSAs were being shared/posted. The purpose of this question is to understand where consumers would have to go to in order to be exposed to these PSA messages. Google Images was particularly useful in addressing this question because in addition to providing the PSA, it also provided the link to any websites that currently hosted it. The PSAs in the sample of this study were available from The American Cancer Society, American Academy of Dermatology, The Melanoma Foundation, The Skin Cancer Foundation, Sun Smart, The World Skin Cancer Foundation, and a preventative treatment website sponsored by the Australian government at http://www.cancer.org.au. As such, all of the PSAs were posted exclusively on government sponsored websites.

### 3.3. Study 2 Discussion

Overall, the majority of PSAs depicted only Caucasian females less than 42 years old. None of the PSAs feature athletes, people working outside, or any ethnicity other than Caucasian. Furthermore, 87.2% of the PSAs messages targeted the tanner and 58.8% of the tanner targeted PSAs were presented as relevant only to young, Caucasian women. The results of this study imply that a range of ages and ethnicities are being ignored in skin cancer PSA construction. Additionally, the PSAs promote preventative skin care behavior at leisure, but not at work or during athletic activities.

## 4. Discussion

African Americans, farmers, and outdoor athletes self-identified as subcultures whose cultural communication creates healthcare norms that leave individuals unaware of the value and necessity of preventative skin care behaviors. The fact that these same groups were not present in a year's worth of PSAs is unlikely a coincidence and more likely representative of the norm in construction of these PSAs. It is of particular concern that only Caucasians are being depicted in these studies, especially considering that African Americans die more frequently of melanoma than Caucasians [21]. Skin cancer is the most preventable form of cancer, as such it is a tragedy for individuals to succumb to the disease because they are unaware of simple preventative steps. The target range of PSAs must be expanded to include unrepresented demographics as well as occupations and activities that are associated with risky sun exposure behaviors. If African Americans, farmers, and outdoor athletes remain unaware of their skin cancer risks, such information needs to be communicated to these groups.

Equally concerning as the limited target audience depicted, was the limited media range through which the PSAs were shared. The PSAs were only available through government sponsored websites dedicated to skin cancer prevention. Given that PSAs are created through government funding, their presence on these websites was not surprising. What is concerning, however, about the PSAs being available *only* through such sites is the limited exposure to the public. People do not look up information related to skin cancer until the topic is salient to them [29]; thus, these websites are pursued by individuals who are looking for information about treating cancer, not preventing it. Because the purpose of these PSAs is to spread the preventative message to those who still have time to take precautions, the dissemination of these messages needs to change so that the message can get to the public before a problem exists. Given that consumers will not go looking for this preventative information, it must be *strategically* placed where they might stumble upon it.

## 5. Conclusion

In short, preventative sun exposure PSAs are available, but the groups most at risk of skin cancer deaths are being ignored in the pictorial element. Additionally, the PSAs are not being distributed to the target audience. To effectively communicate the preventative messages to individuals who need it, future PSAs need to be designed to include at risk groups and then disseminated among multiple media channels.
